# Altitude and Its Association with Low Birth Weight among Children of 151,873 Peruvian Women: A Pooled Analysis of a Nationally Representative Survey

**DOI:** 10.3390/ijerph20021411

**Published:** 2023-01-12

**Authors:** Akram Hernández-Vásquez, Alicia Bartra Reátegui, Rodrigo Vargas-Fernández

**Affiliations:** 1 Centro de Excelencia en Investigaciones Económicas y Sociales en Salud, Vicerrectorado de Investigación, Universidad San Ignacio de Loyola, Lima 15024, Peru; 2 Vicerrectorado de Investigación, Universidad Nacional de San Martín, Tarapoto 22201, Peru; 3 Faculty of Health Sciences, Universidad Científica del Sur., Lima 15067, Peru

**Keywords:** low birth weight, altitude, epidemiologic studies, child and maternal health, Peru

## Abstract

The aim of this study was to determine the relationship between the altitude of residence and the low birth weight (LBW) of the children of pregnant Peruvian women using a nationally representative database. An analysis of individual-level data from the last 13 years (from 2009 to 2021) of the Demographic and Family Health Survey was performed. The outcome variable was LBW, defined as birth weight less than 2500 g, while the independent variable was the altitude of residence in meters above sea level (masl). To estimate the association between the two variables, the crude and adjusted generalized linear model of the Poisson family with a log link was used along with crude and adjusted prevalence ratios, which were estimated with their respective 95% confidence interval. A total of 151,873 women aged 15–49 years were included between 2009 and 2021. The pooled proportion of LBW was 7.0%. As the main finding, the children of mothers residing at an altitude from 2500 to 3499 masl and ≥3500 masl had a higher probability of LBW. It was found that the children of mothers residing at an altitude above 2500 masl were more likely to have LBW. Our results will help to strengthen the cultural practice of maternal health care and increase its coverage in women residing in high-altitude regions.

## 1. Introduction

According to the World Health Organization (WHO), low birth weight (LBW) is a public health indicator that encompasses maternal health and nutritional problems, deficiencies in health care during pregnancy, and socioeconomic conditions [1]. LBW is defined as the first weight recorded immediately after birth that is less than 2500 g, regardless of gestational age [2], and leads newborns to have an increased risk of morbidity and mortality and the onset of cognitive, motor, and language impairments, as well as lifelong consequences [3,4,5,6]. Despite the reduction in this pathology being a global nutrition target for the year 2025 [7], it is estimated that 14.6% of newborns had LBW in 2015, with the highest prevalence being found in developing countries [8]. In Latin America and the Caribbean (LAC), the prevalence of LBW is 10%, and countries such as Haiti (23%) and Guyana (16%) have the highest prevalence in the region [9]. In Peru, the prevalence of LBW is similar to the average for the LAC region (around 10%) [9], and this condition is one of the main indicators of maternal and perinatal problems.

The biomedical literature suggests that factors such as fetal sex and maternal and socioeconomic characteristics can influence the presentation of LBW in a newborn [4,10,11,12,13]. In addition, some recent epidemiological studies suggest that the altitude of residence could be another important determinant for the appearance of LBW in fetuses [14,15]. A recent meta-analysis that evaluated the effect of the altitude of residence and LBW reported that there was an inversely proportional relationship between the two variables, showing an average decrease of 97 g in newborn weight for every thousand-meter increase in the altitude of maternal residences [14]. Although the relationship between altitude and LBW is not well established [16], this association could be influenced by plausible biological pathways that occurred at high altitudes and were based on the exposure of women to hypobaric hypoxia [17,18], low glucose concentrations [19], and an epigenetic process that could limit the intrauterine growth of the fetus [20,21]. Specifically, most epidemiological studies evaluating this relationship were conducted in regions 2000 m above sea level (masl), with similar sociodemographic and geographic characteristics [17,22,23,24].

In Peru, high-altitude regions (Andes region) are characterized by their location in rural areas and at an altitude exceeding 2500 masl [25]. In these regions, pregnant women are not only exposed to hypobaric hypoxia conditions but also to limited and inadequate access to maternal health services and poor conditions [26,27,28,29]. Although there are epidemiological studies that have evaluated the relationship between LBW and the altitude of residence in a Peruvian context at the individual level, these studies were conducted in specific regions of the Andes region of Peru, and therefore, the results cannot be extrapolated to all Peruvian pregnant women residing at high altitudes [30,31,32,33]. Therefore, the aim of the present study was to determine the relationship between the altitude of residence in Peruvian women and LBW using a nationally representative database.

## 2. Materials and Methods

### 2.1. Study Design and Data Sources

The present study used individual-level data from the Demographic and Family Health Survey (ENDES—acronym in Spanish) conducted between 2009 and 2021 (available at http://iinei.inei.gob.pe/microdatos/, accessed on 20 November 2022). The 2009 ENDES was used because the survey has been conducted annually since that year. Briefly, the ENDES is a nationally representative survey that uses a complex sampling design and collects information on sociodemographic and health indicators of women of childbearing age and their children under 5 years of age. The sampling methodology used in the ENDES is provided in detail in the technical reports of the survey [34,35,36,37,38,39,40,41,42,43,44,45,46].

### 2.2. Sampling Design and Sample Size

The sampling design was two-stage, with a sampling frame in each of the stages of the selection of the sampling units (the census for the first stage and the mapping of dwellings for the second stage) [34,35,36,37,38,39,40,41,42,43,44,45,46]. The primary sampling unit (PSU) is represented by the conglomerate made up of one or several blocks that together have between 120 and 140 private dwellings on average for the urban area, and the secondary sampling unit (SSU) includes private dwellings [34,35,36,37,38,39,40,41,42,43,44,45,46]. In the rural area, the PSU is of two types, including the conglomerate of 120 private dwellings on average and the rural census area, which is made up of several population centers that together have between 120 and 140 private dwellings on average; likewise, the SSU is made up of private dwellings [34,35,36,37,38,39,40,41,42,43,44,45,46].

Women selected for the individual questionnaire for women of childbearing age were included in this study. The individual questionnaire collected information on demographic and social characteristics, reproductive history, contraceptive use, maternal and child health, fertility, and domestic violence, among the main topics [34,35,36,37,38,39,40,41,42,43,44,45,46]. Likewise, data from the household questionnaire were used to include basic characteristics and the location of the dwelling [34,35,36,37,38,39,40,41,42,43,44,45,46]. Of the total number of eligible women, a response rate of 98.4% was obtained in 2009 and 95.0% in 2021 [34,35,36,37,38,39,40,41,42,43,44,45,46].

The study sample included women of childbearing age between 15 and 49 years who had had a child within the last five years at the time of the survey. Likewise, women with a residence time of 5 years or more were included. Women who did not reside in the altitude cluster at the time of pregnancy were excluded.

### 2.3. Variables

#### 2.3.1. Outcome Measure

The outcome variable of this study was LBW coded as “1” if the mother reported that the child was born weighing less than 2500 g and “0” otherwise. This classification was based on the definition given by the WHO [2].

#### 2.3.2. Exposure

The exposure variable was the altitude of the cluster where the dwelling was located. In the ENDES, specifically since 2015, the measurement of the altitude was given in masl using an application to obtain georeferenced data (latitude, longitude, altitude) through a global positioning system measured at one meter from the front door of the respondent’s home [47,48,49,50,51,52]. The altitude was classified as: <1500; 1500 to 2499, 2500 to 3499, or 3500 or more masl based on the definition of altitude described by Barry and Pollard [53].

#### 2.3.3. Covariates

Covariates were selected based on previous studies and data available in the ENDES [22,33,54]. Thus, we included the age group of the mother at delivery (from 15 to 24, 25 to 34, 35 to 49 years), the sex of the child (female, male), nulliparity (no, yes), wealth quintile (quintile 1 [Q1 poorest], Q2, Q3, Q4, Q5 [richest]), and area of residence (urban, rural).

### 2.4. Statistical Analysis

A combined database of the 13 years (from 2009 to 2021) of the ENDES was constructed and used for analysis. We used Stata version 17.0 (StataCorp) for data management and analysis. Prior to any statistical analysis, the data were weighed using the sampling weight, PSU, and strata to consider the representativeness and sampling design of the ENDES by using the ‘svy’ command. To specify the sample design, a strata and cluster identifier was created for each year of the ENDES, and the sample weight was divided by 1,000,000.

Initially, a descriptive analysis was performed to report the characteristics of the participants, including the frequency of LBW, the frequency of women by their altitude of residence, and the covariates of interest using absolute and relative frequencies for each year which were pooled. Next, the crude and adjusted generalized linear model of the Poisson family with a log link was used to evaluate the association between LBW and the altitude of residence. The association results are expressed as prevalence ratios (PR) and their 95% confidence intervals (CI). The possible problem of multicollinearity was evaluated by performing a variance inflation factor test which found no correlation between the independent variable and covariates. A *p*-value of <0.05 was considered statistically significant.

### 2.5. Ethical Considerations

Approval from a research ethics committee was not sought for this retrospective study because only anonymized secondary data that are in the public domain and available at http://iinei.inei.gob.pe/microdatos/ (accessed on 20 November 2022) were used. The process of downloading and using the ENDES databases is described in the study by Hernández-Vásquez and Chacón-Torrico [55]. ENDES participants gave informed consent before participating in the survey.

## 3. Results

### 3.1. Study Population

A total of 151,873 women aged 15–49 years were included between 2009 and 2021. Of the total participants included, 51.3% of the children were male, and 47.6% of the mothers belonged to the 25–34 age group. In addition, 26.3% of the mothers were nulliparous, and most of the mothers belonged to the middle (22.1%) and poor (21.8%) wealth quintiles and resided in an urban area (76.4%). On the other hand, the highest proportions of LBW were observed in the years 2010 (8.0%), 2013 (7.9%), and 2015 (7.5%), while the lowest proportions were found in the years 2014 (6.2%), 2019 (6.2%), and 2021 (6.5%). Further details of the characteristics of the population included according to the year of the survey are shown in Table 1.

### 3.2. Sociodemographic Characteristics of Women and Children according to Low Birth Weight

Overall, the pooled proportion of LBW between 2009 and 2021 was 7.0%. Likewise, the highest prevalence of LBW was observed in female children (7.4%) whose mothers belonged to the 15–24 age group (7.8%) and who were their first child (7.6%). Additionally, mothers residing between 3500 and 3499 masl (9.0%), belonging to the poorest wealth quintile (9.3%), and in a rural area (8.5%) had the highest proportion of children with LBW (Table 2).

### 3.3. Sociodemographic Characteristics of Women and Children according to the Mother’s Altitude of Residence

On average, we found that 71.6% of the mothers resided at an altitude lower than 1500 masl, followed by 14.0%, 7.9%, and 6.4% of the mothers residing at an altitude from 2500 to 3499 masl, ≥3500 masl, and from 1500 to 2499 masl; respectively (Table 1). Regarding the proportion of LBW according to the altitude of residence, we observed that at an altitude ranging from 0 to 2499 masl, a higher proportion of children weighed 2500 gr or higher, while at an altitude higher than 2499 masl, children were identified as having a higher proportion of LBW compared to a weight of 2500 gr or higher (Table 3). Regarding the sociodemographic characteristics and according to an altitude of residence above 3499 masl, it was found that the majority of children were male (8.0%), the mothers belonged to the age group of 35–49 years (8.0%), it was their second or older child (8.4%), and they belonged to the poorest wealth quintile (19.9%) and resided in a rural area (20.9%) (Table 3).

### 3.4. Association between Low Birth Weight and Mother’s Altitude of Residence

In the crude model, the LBW was associated with an altitude of residence from 2500 to 3499 masl and ≥3500 masl. Likewise, the same pattern was found in both the adjusted and the crude models. Specifically, model 1, which was adjusted for the mother’s age, child’s sex, wealth quintile, nulliparity, and area of residence, found that the children of mothers residing at an altitude between 2500 and 3499 masl (adjusted PR [aPR]: 1.25, 95% CI: 1.17–1.34) and ≥3500 masl (aPR: 1.15, 95% CI: 1.06–1.24) had a higher probability of having LBW, while in model 2, when adjusted for all variables in model 1 and the survey year, the same pattern was identified, with the children of mothers residing at residence altitudes of 2500–3499 masl (aPR: 1.25, 95% CI: 1.16–1.34) and ≥3500 masl (aPR: 1.14, 95% CI: 1.05–1.24) demonstrating a higher probability of presenting LBW (Table 4).

The sample weights and sample design of ENDES were included for all the estimates.

Model 1: Model with the age group of the mother, sex of the child, nulliparity, wealth index, and area of residence.

Model 2: Model with the age group of the mother, sex of the child, nulliparity, wealth index, area of residence, and year of the survey.

## 4. Discussion

Our study sought to determine the association between the altitude of residence in Peruvian mothers and the presence of LBW in their children by performing an analysis comprising 13 years of a nationally representative survey. We found the pooled proportion of LBW to be 7% in the study period. As the main finding, we observed that after adjusting for potential confounders, mothers residing at an altitude of 2500–3499 masl and ≥3500 masl had a higher probability of having a child with LBW compared to those residing at an altitude lower than 2500 masl. It should be noted that the temporality of this association was based on the assessment of LBW in the last child under five years of age.

We found that 7% of newborns were found to have LBW in the study period, with LBW ranging from 6.2% in 2014 and 2019 to 8.0% in 2010. This finding is lower than that reported in studies conducted in high-altitude regions of the United States (11.8%) [56] and Nepal (12.9%) [57], but it is higher than that reported in a study conducted in high-altitude regions of India (3.2%) [58], Tibet (3.0%) [59], and Argentina (where the proportion of LBW was 5.6% in males and 5.7% in females) [60]. These dissimilar proportions between regions could be attributed to the geographic, ethnic, economic, and sociodemographic characteristics and the length of residence of the mothers in each high-altitude region, which could determine the difference between the anthropometric measures found in the studies [54,61]. Although the proportion of LBW found in our study remains below that reported in countries located in other high-altitude regions, this finding exposes a public health problem for the Peruvian health system. Specifically, LBW is a pathology associated with infant mortality in low- and high-income countries, and it has also been reported that countries with high infant mortality have a higher prevalence of LBW [62]. In this sense, governmental institutions should follow the WHO recommendations for the adequate control of perinatal outcomes [63], in which access to and compliance with prenatal care is a key determinant for the adequate development of the fetus, especially when high-altitude regions have geographic and cultural barriers that reduce access to maternal health programs.

The identified association between the altitude of maternal residence and LBW has previously been reported in the biomedical literature [14,22,23,33,54]. Specifically, epidemiological studies evaluating this association reported a decrease in LBW with the increasing altitude of maternal residence [14,22,23,33,54]. A recent meta-analysis that systematized the findings of 52 studies conducted in high-altitude regions of the United States, Mexico, Peru, Bolivia, New Guinea, Tibet, Kyrgyzstan, Nepal, China, Saudi Arabia, Italy, and England reported a decrease of almost 100 g for every thousand-meter increase in the altitude of maternal residence [14]. Additionally, previous studies suggest that the decrease in birth weight occurs at an altitude exceeding 2000 masl, at which the barometric pressure results in a hypoxic effect, similar to what was reported in our study [16,31]. The association reported in our study could be attributed to different biological pathways that develop from hypobaric hypoxia [17]. One of these pathways is related to reduced fetal glucose supply and consumption, which is favored by increased peripheral insulin sensitivity at high altitudes [64,65,66], with subtle changes in the insulin-like growth factor axis being related to intrauterine fetal restraint [67]. In addition, previous studies indicate that the relationship between LBW and the altitude of residence depends on the time of adaptation of the pregnant woman to high altitudes, with women who have a shorter stay in high altitude regions demonstrating higher LBW levels in their children [31]. This difference could be due to genetic or epigenetic changes that occur as a compensatory mechanism at high altitudes, with pregnant women experiencing hematological (increased hematocrit and erythropoietin) and cardiovascular (increased placental vascularization) changes that are transferred to the developing fetus [17]. In this sense, the main strategies that seek to reduce the impact of LBW in the Peruvian territory should identify this association in order to achieve greater coverage of maternal health care in high-altitude regions, especially when more than five million people live in these regions and infant mortality ranges at between 50 and 60 deaths per 1000 live births [25,68].

Our results could have an impact on clinical practice and public health. In clinical practice, the association between the altitude of residence and LBW observed in our study could allow health personnel to better follow and comply with prenatal check-ups of pregnant Peruvian women residing at a high altitude (>2500 masl). On the other hand, maternal health care that seeks to reduce negative perinatal outcomes in rural or high-altitude regions should implement an intercultural approach since native women (Aymara or Quechua) have ancestral practices that generate a negative perception of maternal health care, resulting in a higher frequency of home births attended by midwives and lower use of formal health services [27]. In addition, Peruvian government programs should prioritize strategies that seek to achieve greater maternal healthcare coverage in high-altitude areas, which are characterized by geographic barriers and poor conditions. This could reduce negative maternal and perinatal outcomes, in addition to reducing the short- and long-term consequences of LBW.

Our study has limitations and strengths. First, one of the main limitations is the cross-sectional design of our study, which prevents establishing causality between the variables of interest due to a lack of temporality in their measurement. Therefore, longitudinal studies are required to determine the causality between the exposure variable (altitude of residence) and the outcome variable (LBW). Second, as birth weight is a variable self-reported by the mother, there could be a recall bias or inaccuracy in the data since the interviewer requests the exact amount of the newborn’s weight from the last pregnancy in the last five years. Third, by using different years of the ENDES, different sample frames were obtained that could have generated differences in the characteristics of the mother and child between each year, and this may have been more notable in the 2020 and 2021 ENDES, which collected part of the information by telephone due to the COVID-19 pandemic [45,46]. Fourth, due to confidentiality issues, the altitude of residence is reported in the ENDES databases at the cluster level and not specifically at the household level. Finally, there is a risk of residual confounding due to the impossibility of measuring potential confounders such as risk factors in pregnancy (tobacco use), clinical variables (pregnancy-related diseases and congenital conditions in the newborn), and nutritional variables (type and amount of food consumed by the woman during gestation) because of data availability. On the other hand, gestational age could not be included as a covariate because the ENDES only provides information on the duration of the current pregnancy (in months) for each year of the survey, which is equivalent to a small number of participants. In addition, gestational age is not a variable that enters into the definition of low birth weight and therefore does not affect current estimates. However, our study used a nationally representative database conducted under the DHS program format and considered more than 150,000 women with children over a 13-year time horizon which allowed the relationship between the variables of interest to be determined. The interviewers of this survey had been duly trained to obtain the data, thereby increasing the reliability of the variables.

## 5. Conclusions

In conclusion, it was found that mothers residing at an altitude above 2500 masl were more likely to have children with LBW. In this sense, the findings of this study will allow the development of strategies that include the cultural practices of women and ensure greater coverage and control of pregnant women to reduce the prevalence and consequences of LBW throughout the life of the newborn.

## Figures and Tables

**Table 1:** Characteristics of the population included according to the year of the survey.

	2009–2021	2009	2010	2011	2012	2013	2014	2015	2016	2017	2018	2019	2020	2021
Characteristics	%	%	%	%	%	%	%	%	%	%	%	%	%	%
Overall (*n*)	151,873	6988	6271	6196	6796	6488	6870	17,437	15,478	15,628	17,398	15,608	14,108	16,607
Low birth weight														
No	93.0	93.2	92.0	93.4	93.0	92.1	93.8	92.5	93.1	92.9	92.9	93.8	93.4	93.5
Yes	7.0	6.8	8.0	6.6	7.0	7.9	6.2	7.5	6.9	7.1	7.1	6.2	6.6	6.5
Altitude (in meters above sea level)														
<1500	71.6	65.5	64.8	67.5	67.5	72.8	72.2	73.2	73.8	74.9	73.4	73.7	73.9	73.5
1500–2499	6.4	6.6	7.2	6.6	7.3	6.8	5.6	6.5	6.5	6.6	6.1	5.8	5.2	6.7
2500–3499	14.0	16.2	17.3	15.4	15.7	13.3	13.8	12.8	12.9	12.1	13.7	14.0	14.8	12.7
≥3500	7.9	11.7	10.7	10.5	9.6	7.1	8.4	7.6	6.8	6.5	6.8	6.5	6.1	7.1
Age group of mother (in years)														
35–49	21.3	23.3	24.2	22.6	24.6	23.1	22.3	20.6	20.5	21.5	19.7	19.7	18.3	18.7
25–34	47.6	48.1	47.2	47.7	46.8	47.6	46.9	47.8	48.5	47.7	47.7	47.2	48.0	47.4
15–24	31.1	28.6	28.6	29.7	28.7	29.2	30.8	31.6	31.1	30.8	32.6	33.0	33.6	33.9
Sex of child														
Female	48.7	47.4	49.1	48.7	48.0	47.6	48.9	48.9	48.1	49.8	48.5	49.5	49.0	49.3
Male	51.3	52.6	50.9	51.3	52.0	52.4	51.1	51.1	51.9	50.2	51.5	50.5	51.0	50.7
Wealth index														
Q1 (poorest)	19.3	19.1	19.5	19.7	18.8	19.2	18.8	18.3	17.2	18.3	20.4	20.6	20.7	20.6
Q2	21.8	21.2	21.3	19.1	22.1	21.9	22.6	20.5	21.8	22.9	22.1	22.5	23.6	21.9
Q3	22.1	22.2	22.7	24.7	23.5	22.9	22.9	21.4	22.4	21.7	21.0	20.7	21.3	21.3
Q4	20.0	20.8	21.0	20.8	19.8	20.1	20.4	20.4	20.0	20.2	19.4	19.1	18.0	20.0
Q5 (richest)	16.8	16.7	15.4	15.7	15.7	15.8	15.3	19.4	18.6	16.9	17.1	17.0	16.5	16.2
Nulliparity														
No	73.7	75.0	74.5	74.4	75.0	73.4	73.4	73.7	74.0	71.3	74.0	73.3	73.6	73.3
Yes	26.3	25.0	25.5	25.6	25.0	26.6	26.6	26.3	26.0	28.7	26.0	26.7	26.4	26.7
Area of residence														
Urban	76.4	71.1	70.6	72.1	71.6	74.7	75.7	78.6	79.0	79.7	78.8	78.5	78.1	79.4
Rural	23.6	28.9	29.4	27.9	28.4	25.3	24.3	21.4	21.0	20.3	21.2	21.5	21.9	20.6

%: weight proportion, the sample weights and sample design of ENDES were included.

**Table 2:** Characteristics of the participants according to low birth weight.

Characteristics	Low Birth Weight		
	No	Yes	*p*-Value *
	*n* (%)	*n* (%)	
Overall	141,262 (93.0)	10,611 (7.0)	
Altitude (in meters above sea level)			
<1500	92,300 (93.6)	6273 (6.4)	<0.001
1500–2499	11,140 (93.5)	753 (6.5)	
2500–3499	23,244 (91.0)	2119 (9.0)	
≥3500	14,578 (91.6)	1466 (8.4)	
Age group of mother (in years)			
35–49	30,227 (92.6)	2498 (7.4)	<0.001
25–34	67,526 (93.8)	4438 (6.2)	
15–24	43,509 (92.2)	3675 (7.8)	
Sex of child			
Female	68,917 (92.6)	5575 (7.4)	<0.001
Male	72,345 (93.5)	5036 (6.5)	
Wealth index			
Q1 (poorest)	34,012 (90.7)	3318 (9.3)	<0.001
Q2	36,233 (93.2)	2635 (6.8)	
Q3	30,781 (93.7)	2065 (6.3)	
Q4	23,863 (93.9)	1549 (6.1)	
Q5 (richest)	16,373 (93.7)	1044 (6.3)	
Nulliparity			
No	105,483 (93.3)	7732 (6.7)	<0.001
Yes	35,779 (92.4)	2879 (7.6)	
Area of residence			
Urban	101,053 (93.5)	7043 (6.5)	<0.001
Rural	40,209 (91.5)	3568 (8.5)	

%: weight proportion, the sample weights and sample design of ENDES were included, * Estimated *p*-value using the Chi-square test with Rao–Scott adjustment.

**Table 3:** Characteristics of the participants according to altitude of residence.

Characteristics	Altitude of Residence *				
	<1500	1500–2499	2500–3499	≥3500	*p*-Value **
	*n* (%)	*n* (%)	*n* (%)	*n* (%)	
Low birth weight					
No	92,300 (72)	11,140 (6.4)	23,244 (13.7)	14,578 (7.8)	<0.001
Yes	6273 (66.3)	753 (5.9)	2119 (18.1)	1466 (9.6)	
Age group of mother (in years)					
35–49	22,137 (73)	2294 (5.9)	4905 (13.2)	3389 (8)	<0.001
25–34	47,125 (72)	5644 (6.3)	11,694 (13.8)	7501 (7.9)	
15–24	29,311 (70.1)	3955 (6.9)	8764 (15.1)	5154 (7.9)	
Sex of child					
Female	48,340 (71.5)	5798 (6.4)	12,507 (14.2)	7847 (7.8)	0.269
Male	50,233 (71.7)	6095 (6.4)	12,856 (13.9)	8197 (8)	
Wealth index					
Q1 (poorest)	14,448 (39.8)	3665 (11)	11,095 (29.3)	8122 (19.9)	<0.001
Q2	24,953 (66.4)	2661 (5.5)	6927 (17.6)	4327 (10.5)	
Q3	24,796 (79.5)	2090 (4.9)	3763 (10.6)	2197 (5)	
Q4	20,178 (85)	1900 (5.3)	2256 (7.2)	1078 (2.5)	
Q5 (richest)	14,198 (88.6)	1577 (5.6)	1322 (4.7)	320 (1)	
Nulliparity					
No	72,667 (70.7)	8866 (6.5)	19,203 (14.3)	12,479 (8.4)	<0.001
Yes	25,906 (74.1)	3027 (6.1)	6160 (13.2)	3565 (6.6)	
Area of residence					
Urban	82,555 (82.3)	6921 (4.7)	12,386 (9.1)	6234 (3.9)	<0.001
Rural	16,018 (37.2)	4972 (11.9)	12,977 (30.1)	9810 (20.9)	

%: weight proportion, the sample weights and sample design of ENDES were included, * In meters above sea level, ** Estimated *p*-value using the Chi-square test with Rao–Scott adjustment.

**Table 4:** Association between altitude of residence and low birth weight.

	Crude Model		Model 1		Model 2	
Variable	PR (95% CI)	*p*-Value	PR (95% CI)	*p*-Value	PR (95% CI)	*p*-Value
Altitude *						
<1500	Ref.		Ref.			
1500–2499	1.00 (0.91–1.10)	0.954	0.93 (0.84–1.02)	0.124	0.92 (0.84–1.02)	0.101
2500–3499	1.39 (1.31–1.49)	<0.001	1.25 (1.17–1.34)	<0.001	1.25 (1.16–1.34)	<0.001
≥3500	1.31 (1.21–1.41)	<0.001	1.15 (1.06–1.24)	0.001	1.14 (1.05–1.24)	0.001

PR—prevalence ratio, CI—confidence interval, Ref—reference category, * In meters above sea level.

## Data Availability

Not applicable.
